# Aortoarteritis and cardiomyopathy in a child with Blau Syndrome

**DOI:** 10.1186/1546-0096-9-S1-P36

**Published:** 2011-09-14

**Authors:** RP Khubchandani, RP Hasija, I Touitou, C Khemani

**Affiliations:** 1 Pediatric Rheumatology Clinic, Jaslok Hospital, Mumbai, India; 2 Pediatric Rheumatology Clinic, Jaslok Hospital, Mumbai, India; 3 University Hospital Center of Montpellier, Montpellier Cedex 5,France; 4 Pediatric Rheumatology Clinic, Jaslok Hospital, Mumbai, India

## Background

Blau Syndrome, transmitted as an autosomal dominant condition, has been shown to have a novel mutation in the CARD15 (NOD2) locus. Cardiovascular features are rarely described in Blau Syndrome.

## Aim

To report rare cardiovascular manifestations in a child with Blau Syndrome.

## Method

Retrospective chart review of a child whom we first saw in 2002 and have been following up regularly since 2008.

## Description

MG, now a 10 year old female, was seen with a history of recurrent fevers since 1 month of life with bilateral knee effusions noted at 1.5 years of age, in the year 2002. This was initially treated as ANA negative oligoarticular JIA, requiring intra-articular steroid injections twice. She has never had a history of skin manifestations or uveitis. Over time, the knees showed increasing bogginess without restriction of activity.

Six years after being lost to follow up, in mid 2008, at the age of 8 years, she presented with features of cardiac failure, arrhythmias (ventricular premature beats) and arthritis of the elbow joint. Investigations showed normal blood counts, normal eye examination and raised ESR. The 2D Echo showed dilated cardiomyopathy with a 20% ejection fraction with an abnormal myocardial echogenicity. Recurrent fevers since early childhood with arthritis and cardiomyopathy lead to a suspicion of an autoinflammatory syndrome. Further investigations showed a normal serum ACE and the DNA analysis showed a punctual mutation at G464W in exon 4 of the CARD15 gene. Liver and cardiac biopsies were deferred due to poor cardiac function. An identical genetic mutation was found in the mother who had a history of recurrent fevers, abdominal pain and respiratory symptoms that had never been investigated. Since then, MG has been on tapering oral steroid (started at 1mg/kg/day), injectable Methotrexate (15- 17.5 mg/m^2^) and anti-failure line of therapy. Initially, her symptoms improved with only a marginal improvement in the cardiac function. In early 2009, mild hypertension was documented and in September 2009, carotid pulsations were found reduced. By mid 2010, she had severe hypertension (230/120 mmHg), uncontrolled on multiple anti-hypertensives, with renal and carotid bruits. Digital Subtraction Angiography showed generalized narrowing of the aorta, carotids, left subclavian artery and bilateral renal arteries, suggestive of an aortoarteritis. The cerebral circulation was maintained normally with diffuse collaterals. The ultrasound abdomen showed a contracted left kidney with the right kidney perfusion maintained with an accessory artery with multiple renal hypo-echogenecities. The renal function and calcium excretion were normal with negative ANCAs. Three years into follow-up after diagnosis of aortoarteritis, on continuing therapy, she remains asymptomatic with an improvement in her cardiac function to an ejection fraction of 55%. Intervention such as stenting of the stenosed major vessels has currently been deferred due to the widespread nature of the aortoarteritis being aided with diffuse collateral circulation maintaining normal function.

## Conclusions

In this child we never saw the classic triad of rash, arthritis and uveitis. Cardiomyopathy and aortoarteritis were the unusual manifestations which have been very rarely reported worldwide in Blau Syndrome patients.

